# Predicting the seasonal distribution of fall armyworm in North America using species distribution models

**DOI:** 10.1002/ps.70217

**Published:** 2025-09-18

**Authors:** Fan‐Qi Gao, Robert L Meagher, Rodney N Nagoshi, Jason W Chapman, Regan Early

**Affiliations:** ^1^ Centre for Ecology and Conservation University of Exeter Cornwall UK; ^2^ Center for Medical, Agricultural and Veterinary Entomology, Agricultural Research Service, US Department of Agriculture Gainesville FL USA; ^3^ Department of Entomology Nanjing Agricultural University Nanjing China

**Keywords:** environmental niche models, insect migration, realized niche, seasonal habitat, *Spodoptera frugiperda*

## Abstract

**BACKGROUND:**

Species distribution models (SDMs) are widely used in pest management to predict outbreak areas. Migratory pests cause seasonal crop damage through long‐distance migration, making it crucial to understand their seasonal activity when estimating outbreak regions. Fall armyworm (FAW*)*, a highly migratory pest, was studied using SDMs to predict its seasonal distribution in the central and eastern USA and explore the environmental factors influencing its distribution.

**RESULTS:**

We used monthly environmental and species distribution data to model each month or season individually. Based on model results, the suitable habitat for FAW expands rapidly in summer, covering most of the central and eastern USA in July and August, and then begins to contract in September. Based on the environmental variables included in models for different seasons, FAW distribution in winter is mainly influenced by minimum temperature. In spring, rainfed corn cultivation area and Normalized Difference Vegetation Index (NDVI) also play important roles. In summer, minimum temperature is no longer important, and the main factors are precipitation, evapotranspiration, rainfed corn cultivation area, and NDVI. In autumn, minimum temperature becomes important again, while rainfed corn cultivation area is no longer a key factor.

**CONCLUSION:**

This study indicates that FAW may have outbreaks across the central and eastern USA in July and August, emphasizing the necessity of implementing early large‐scale pest control. Using separate models for each season, rather than a single model to predict whether seasonal distribution could improve prediction accuracy by identifying key environmental variables in each season and refining niche characterization. © 2025 The Author(s). *Pest Management Science* published by John Wiley & Sons Ltd on behalf of Society of Chemical Industry. This article has been contributed to by U.S. Government employees and their work is in the public domain in the USA.

## Graphical abstract

The species distribution model predicts fall armyworm habitat expands in summer, covering much of the central and eastern USA in July–August, before contracting again in September.

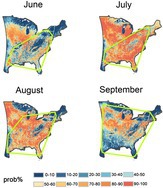

## 1 INTRODUCTION

Global changes due to environmental variability in temperatures are affecting pest populations and causing range expansion of many crop pests toward higher latitudes, posing a threat to agricultural productivity on a broader scale.^1^, ^2^, ^3^, ^4^ Predicting potential pest outbreak areas is crucial for implementing preventive measures. Species Distribution Models (SDMs) are commonly used to project pest outbreaks by establishing relationships between a species' geographic distribution and environmental factors to predict its potential habitats.^5^ Many pests cause seasonal infestations of crops through long‐distance migration, which implies that SDMs assessing habitat suitability on an annual scale cannot accurately describe the range and timing of pest outbreaks.^6^ Despite this, annual SDMs remain the most common method for assessing the distribution of migratory pests.^7^, ^8^, ^9^ Therefore, research should shift towards seasonal SDMs to provide more dynamic and precise information on pest distribution.

The core concept of SDMs is the ecological niche, which refers to the environmental conditions necessary for a species to survive and maintain its population in a specific space.^10^ For many pests exhibiting seasonal infestation patterns, temporary resources are often located in regions with pronounced environmental seasonality (*e.g*., winter/summer in temperate zones or dry/wet seasons in tropical regions). Therefore, the environmental space (ecological niche) occupied by insects in different seasons will be influenced by the prevailing climatic characteristics and phenology, which in turn results in seasonal changes in the environmental variables crucial to species distribution.^6^ For instance, the growth performance of the rice leaf bug *Trigonotylus caelestialium* on a single host plant changes due to the seasonal variation in host quality.^11^ In such cases, species are merely constrained to specific environmental spaces by the different environmental conditions available across seasons, without any actual shift in their ecological niche.

To adapt to seasonal environmental variations, some insects may exhibit phenotypic plasticity to enhance their adaptability in ever‐changing environments, leading to actual niche shifts. For example, fire ants *Solenopsis invicta* exhibit a higher critical thermal maximum in summer compared to other seasons.^12^ Thus, to improve the accuracy of seasonal SDMs, it is necessary to consider the complex impacts of seasonal environmental changes on species distributions. This can be achieved by constructing separate SDMs for different seasons.

The fall armyworm (*Spodoptera frugiperda*, hereafter ‘FAW’) is a highly migratory pest native to the Americas.^13^, ^14^, ^15^ It cannot enter diapause under adverse conditions, limiting its overwintering range to the tropical and subtropical regions of the Americas, with southern Texas and southern Florida being the northernmost areas in the USA where FAW populations can breed year‐round.^13^, ^16^ In spring, FAW migrates northward in search of seasonal resources, infesting most states east of the Rocky Mountains and reaching southern Canada by late summer.^13^, ^17^, ^18^ In addition, some infestations have also been recorded in the western USA.^19^

FAW larvae have been reported to feed on up to 190 plant species in the Americas.^20^ They cause significant damage to major crops such as corn, rice, and sorghum, primarily through direct feeding and the introduction of pathogens during feeding.^21^, ^22^, ^23^ In the USA, annual yield losses caused by FAW are estimated at approximately $300 million, which can rise to $500 million or more during major outbreak years.^23^ Since 2016, FAW has expanded its distribution range through international trade and migration, becoming a significant global pest.^14^

Given the high migratory ability of FAW and its severe impact on crops, many studies have developed SDMs to predict its potential distribution in different regions. At an annual scale, some SDMs have predicted the current global distribution of FAW,^9^, ^24^ while others have focused on specific regions or projected its distribution under future climate conditions.^25^, ^26^, ^27^ At a seasonal scale, a recent study used a single model to predict FAW distribution across different seasons, overlooking the potential variation in how environmental variables might influence FAW distribution in different seasons.^28^ To account for this variation, our study aims to construct separate SDMs for different seasons to predict the dynamic distribution of FAW and investigate whether it undergoes ecological niche shifts across seasons.

The USA is divided into four regions (https://about-the-usa.com/places/regions.htm, accessed 26 March 2025), among which the Midwest, South, and Northeast serve as the native seasonal habitats of FAW and have relatively comprehensive distribution data. In this study, we collectively refer to these three regions as the ‘central and eastern USA’ and focus our analysis on this area. By aligning FAW's geographic distribution with monthly environmental data, we constructed separate SDMs for each season or month to predict the spatiotemporal habitat suitability of FAW and to identify key environmental factors influencing its distribution. Outside of its native range in the Americas, FAW has maintained high migratory capacity and rapidly exhibited seasonal migratory strategies in several newly invaded regions, such as Asia.^29^, ^30^, ^31^, ^32^ This makes it urgent to also develop models capable of predicting FAW seasonal potential distribution in these newly invaded areas. Studying the environmental factors influencing FAW's seasonal distribution in the central and eastern USA may contribute to the development of predictive tools for pest monitoring and early warning on a global scale.

In addition to predicting the spatiotemporal distribution of FAW and identifying the key environmental factors influencing its distribution, we also aim to explore FAW's seasonal ecological niche and address the following questions: (1) Do the key environmental factors that determine FAW distribution and its ecological niche shift across seasons? (2) If the ecological niche shifts, what are the potential causes of this seasonal variation?

## 2 MATERIALS AND METHODS

### 2.1 Environmental data

Before constructing the models, we considered and collected various climate and land cover variables. The climate datasets included monthly average maximum and minimum temperatures (MINTEMP and MAXTEMP, °C), precipitation (PRECIPI, mm), potential evapotranspiration (EVAPO, mm), and vapor pressure deficit (VAPOR, Pa), all sourced from the CHELSA database (https://chelsa-climate.org/) with a resolution of 0.0083 degrees. We obtained the Normalized Difference Vegetation Index (NDVI) dataset from the MODIS database (https://lpdaac.usgs.gov/products/mod13a3cv006/) with a resolution of 1 km. Rainfed corn cultivation area (RFD, 1000 ha per 5‐arc minute grid cell) were derived from the study by Grogan *et al*.,^33^ with a resolution of 0.083 degrees. Due to the lack of precise corn cultivation data, the RFD dataset represents the average cultivation area for each month of 2015 (a total of 12 raster layers). The rest of the datasets we collected cover monthly averages from 2010 to 2018 (a total of 108 raster layers for each dataset). Corn is the primary host plant of FAW, and the climate factors selected in this study have a significant impact on corn growth and the development of FAW.^9^, ^34^, ^35^, ^36^, ^37^ To ensure consistency in processing these environmental variables, we used the ‘terra’ package in R to rescale all raster data to 1 km resolution.^38^

### 2.2 Distribution data

**Figure 1:**
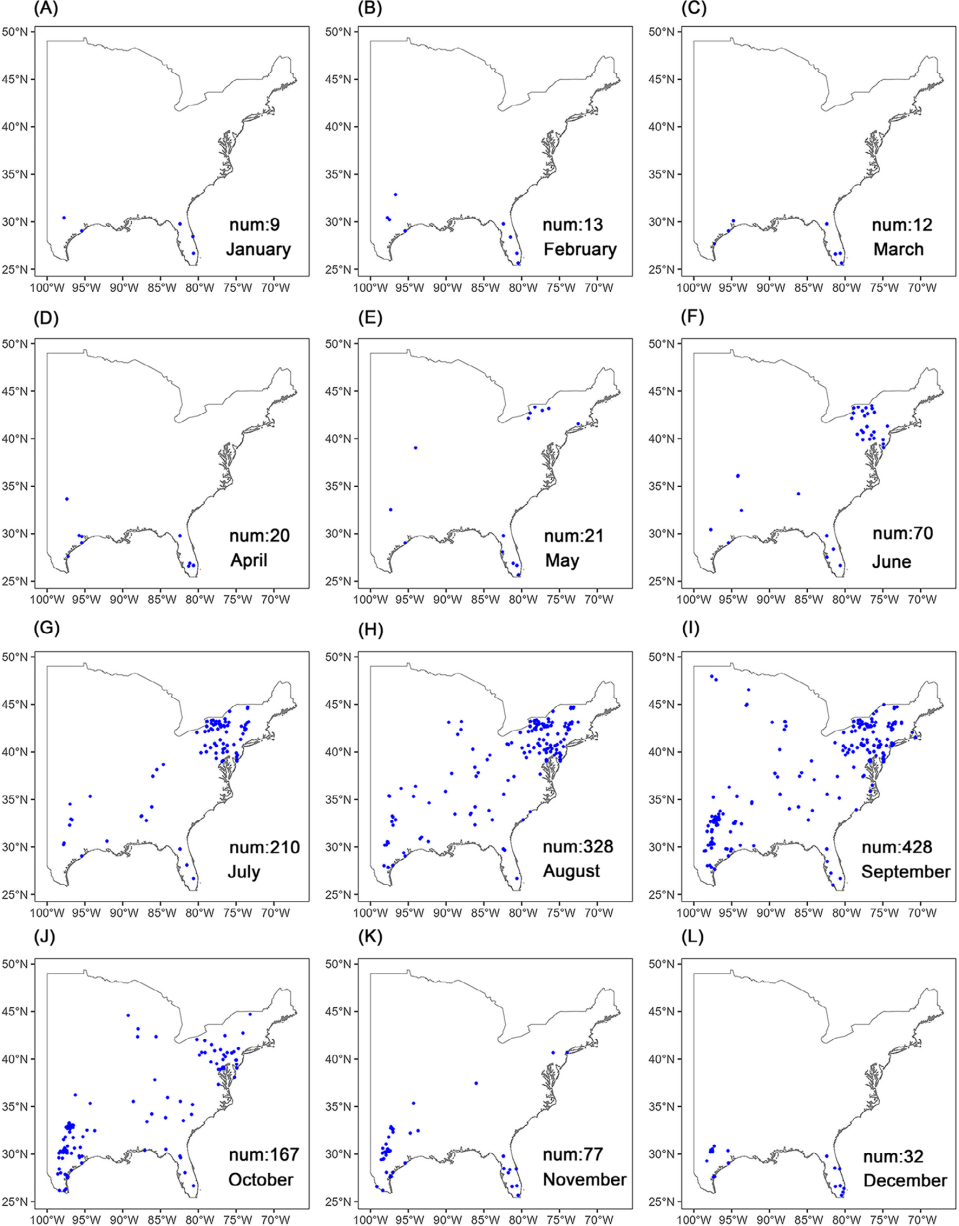
Presence points of fall armyworm (FAW) in the central and eastern USA from 2010 to 2018 used for building the SDMs. Panels represent the presence points from January to December, with ‘num’ indicating the number of presence points for each month.

The distribution records of FAW in the USA from 2010 to 2018 were obtained from the following five sources: (1) Global Biodiversity Information Facility^39^; (2) BAMONA database (https://www.butterfliesandmoths.org/); (3) PestWatch database (http://www.pestwatch.psu.edu/); (4) consultations with managers of USA university agricultural extension programs and relevant researchers; and (5) published papers.^40^, ^41^ The distribution data was filtered to ensure only one occurrence per 1 km grid cell per month per year, resulting in a total of 1387 presence points. Data we have been permitted to share publicly are available in Table S2. After visualizing the presence points, we decided to focus our study on the central and eastern USA, where the majority of data points are located (Fig. 1). Each presence point includes the year and month of the observation and geographical coordinates. We used the ‘terra’ package in R to extract the corresponding environmental information for each presence point from the environmental datasets using latitude and longitude.^38^

### 2.3 Species distribution modelling

We used two methods here for selecting pseudo‐absence points.^42^, ^43^ (1) Random Sampling (RS): The maximum flight distance of individual FAW is approximately 500 km, so we selected pseudo‐absence points within a 500 km range around the presence points.^9^, ^13^, ^44^, ^45^ Using a restricted selection range prevents the model from contrasting completely different climatic conditions and providing an overly simplistic characterization of habitat suitability.^46^, ^47^ Then, within this range, we randomly select a pseudo‐absence point in a grid cell that does not overlap with the presence point, ensuring the number of pseudo‐absence points is equal to the number of presence points. To minimize the likelihood that a randomly selected set of pseudo‐absences might be unrepresentative and bias the model, we generated five sets of pseudo‐absence points. We extracted environmental variables at the locations of all five sets and combined these with presence points to form five datasets, each of which was modelled individually. (2) Random Sampling with Environmental Profiling (RSEP): This method imposes restrictions on the selection range based on RS. For each presence point, we randomly selected 30 background points within 500 km. We then trained a One‐Class Support Vector Machine (OCSVM) classifier with the environmental variables of presence points.^48^ Next, we used this classifier to predict the similarity between the environmental variables of each background point and those of the entire presence dataset, with similarity probabilities ranging from 0 to 1. For each presence point, we randomly select one from the corresponding 30 background points with a similarity probability of 0 as the pseudo‐absence point. As with method 1, we generated five sets of pseudo‐absence points and combined each of them with presence points to form the model datasets.

To construct SDMs, we used Generalized Linear Mixed Models (GLMM), where environmental variables in the model dataset were fixed effects, year was a random effect, and the dependent variable was a binary variable (1/0) corresponding to the presence or pseudo‐absence of FAW. We divided each model dataset into multiple seasonal or monthly subsets. There were too few presence points for some months to model them independently, so we merged data for March, April, and May into a spring dataset, and data for December, January, and February into a winter dataset. We modelled data for the months of June–November individually. Since the RS and RSEP pseudo‐absence methods each generated five model datasets, and each model dataset produced eight sub‐models, a total of 80 models were obtained.

In SDMs, multicollinearity among explanatory variables can lead to model performance instability and prediction uncertainty (Dormann *et al*., 2013). Therefore, we excluded highly correlated variables before constructing the models. We assessed collinearity in the environmental variables for the 80 sub‐models using Pearson correlation and averaged the Pearson correlation coefficients for the same season or month, resulting in eight Pearson matrices. Variables with a correlation coefficient |r| ≥ 0.8, and which were less commonly used in previous FAW SDM studies, were excluded from the model (see Fig. S1). Ultimately, we selected five environmental variables: MINTEMP, PRECIPI, EVAPO, NDVI, and RFD.

Next, we constructed generalized linear mixed models in R using the glmmTMB package with each data subset, incorporating both the linear and quadratic terms of each environmental variable.^49^ We then used the Akaike Information Criterion (AIC) for automated stepwise model selection, where unimportant environmental variables or polynomial terms were removed from the model for each month or season, ultimately resulting in the best model. We evaluated each model using 5‐fold cross validation and assessed the model prediction accuracy on validation data using three parameters: Area Under the Curve (AUC), sensitivity, and specificity. The explanations for these three parameters are as follows: (1) AUC: The area under the receiver operating characteristic (ROC) curve, referred to as AUC, is commonly used as a summary metric to evaluate the performance of a binary classifier. A value of 0.5 represents a random prediction, and values above 0.5 indicate predictions that are better than random. (2) Sensitivity: The proportion of correctly predicted presence cases by the model. (3) Specificity: The proportion of correctly predicted absence cases by the model. To eliminate threshold selection bias on sensitivity and specificity, we employed three threshold selection methods: (1) threshold = 0.5 (If the model's predicted value > = 0.5, it is classified as ‘presence’; otherwise, it is classified as ‘absence’); (2) sensitivity = specificity; (3) sensitivity = 0.95.^50^, ^51^ We compared the predictive capabilities of all models generated using the RS and RSEP pseudo‐absence point selection techniques using AUC, sensitivity, and specificity. RSEP models demonstrated notably higher accuracy (see Results) and were therefore used to predict the seasonal suitable habitats for FAW in the central and eastern USA from 2010 to 2018. To summarize the prediction results, we averaged the monthly predictions across 9 years. Due to higher model performance and greater practical relevance, the raster maps for June to September are presented in the Results section. To differentiate the environmental suitability of various regions, we divided the suitability index evenly into 10 intervals ranging from 0% to 100% and used different colors on the raster maps to represent areas within each suitability index interval. We wanted to determine whether the optimal environmental values for FAW occurrence vary with environmental fluctuations across different months. To do so, for each model used for prediction, we generated the response curves of the environmental variables and extracted the environmental values corresponding to the peaks of the response curves as the optimal environmental values. Then, we averaged the optimal values for all models (made with RSEP method) for the same month (or season) to obtain the optimal environmental values average. We compared the optimal environmental value average to the total environmental value average across the central and eastern USA, and the environmental value average at all presence points for each environmental variable in each month.

### 2.4 Ecological niche comparison between seasons

We asked whether the environmental conditions from which FAW was recorded varied across the course of a year by looking for niche shifts between months/seasons.^52^, ^53^, ^54^ To calculate the environmental conditions available to FAW in each month/season, we established a 500‐km buffer zone around each occurrence point. This distance aligns with the range used for selecting pseudo‐absence points when constructing the SDMs. Using environmental variables consistent with the SDMs, we extracted the monthly environmental data within the buffer zone of each presence point. Data from all years were pooled together as background data for the subsequent analysis. These background data reflect the available environmental space for each month.

We conducted a principal component analysis (PCA) on the background data using the ‘ecospat’ package in R, generating a two‐dimensional summary of the environmental conditions available to FAW.^53^ We mapped the environmental data for presence points (observed ecological niche) from June to September (‘outbreak’ months with better model predictions and more presence points) and other months (‘non‐outbreak’ months with poorer model predictions and fewer presence points) onto this two‐dimensional space. This approach maximizes separation, quantification, and comparison of the climatic and spatial conditions FAW experiences during these two periods.^53^ We calculated the overlap and mismatch in the observed ecological niche in the two time periods on PCA axes. We also visualized the overlap and mismatch of the niche occupancy along each environmental axis separately, to identify the environmental variables most responsible for the niche differentiation.

## 3 RESULTS

### 3.1 Comparison of model predictive accuracy under random and environmentally profiled pseudo‐absence selection methods

**Figure 2:**
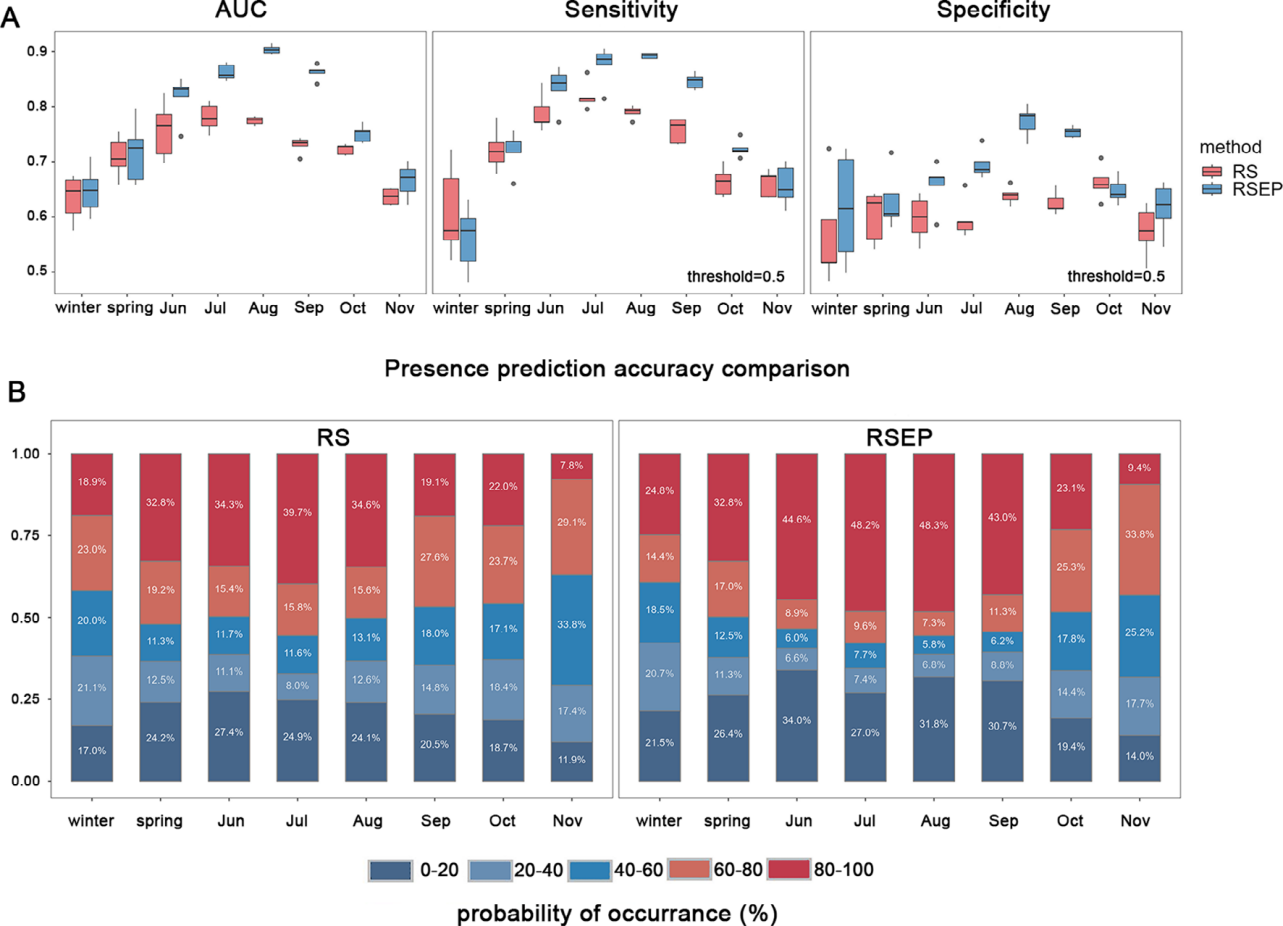
Comparison of the predictive capability of seasonal models constructed using RS and RSEP. (A) Comparison of the Area Under Curve (AUC), sensitivity, and specificity. In the box plots, the ends of the box represent the upper and lower quartiles, the solid line inside the box represents the median, and the whiskers extending from the box represent the maximum and minimum values after excluding outliers. In this figure, the threshold for sensitivity and specificity is set at 0.5. Results using two additional thresholds are shown in Fig. S1(A), (B). (B) Comparison of prediction accuracy at presence points. We divided the probability of occurrence evenly into five intervals between 0% and 100% and calculated the percentage of predicted occurrence values within each interval (labeled in white). Different intervals are represented by different colors.

In models constructed using environmentally‐profiled pseudo‐absences (RSEP), those from June to September exhibited higher predictive performance compared to models from other months (Figs 2(A), S2(A), (B)). Additionally, during this period, RSEP models demonstrated higher predictive performance than those constructed using random pseudo‐absences (RS) (Figs 2(A) and S2(A), (B)), with more presence points predicted to have a probability of FAW occurrence in the highest interval (0.8 to 1.0, Fig. 2(B)). In contrast, during other months, the performance of models constructed using these two methods did not show much difference and overall relatively low predictive power (Figs 2(A), (B) and S2(A), (B)). These results indicate that the advantage of the RSEP method is most pronounced during the peak activity season (when presence records are more comprehensively collected), whereas in other seasons, its advantage diminishes due to the decline in the quality of collected presence data.

### 3.2 Niche comparison between seasons

**Figure 3:**
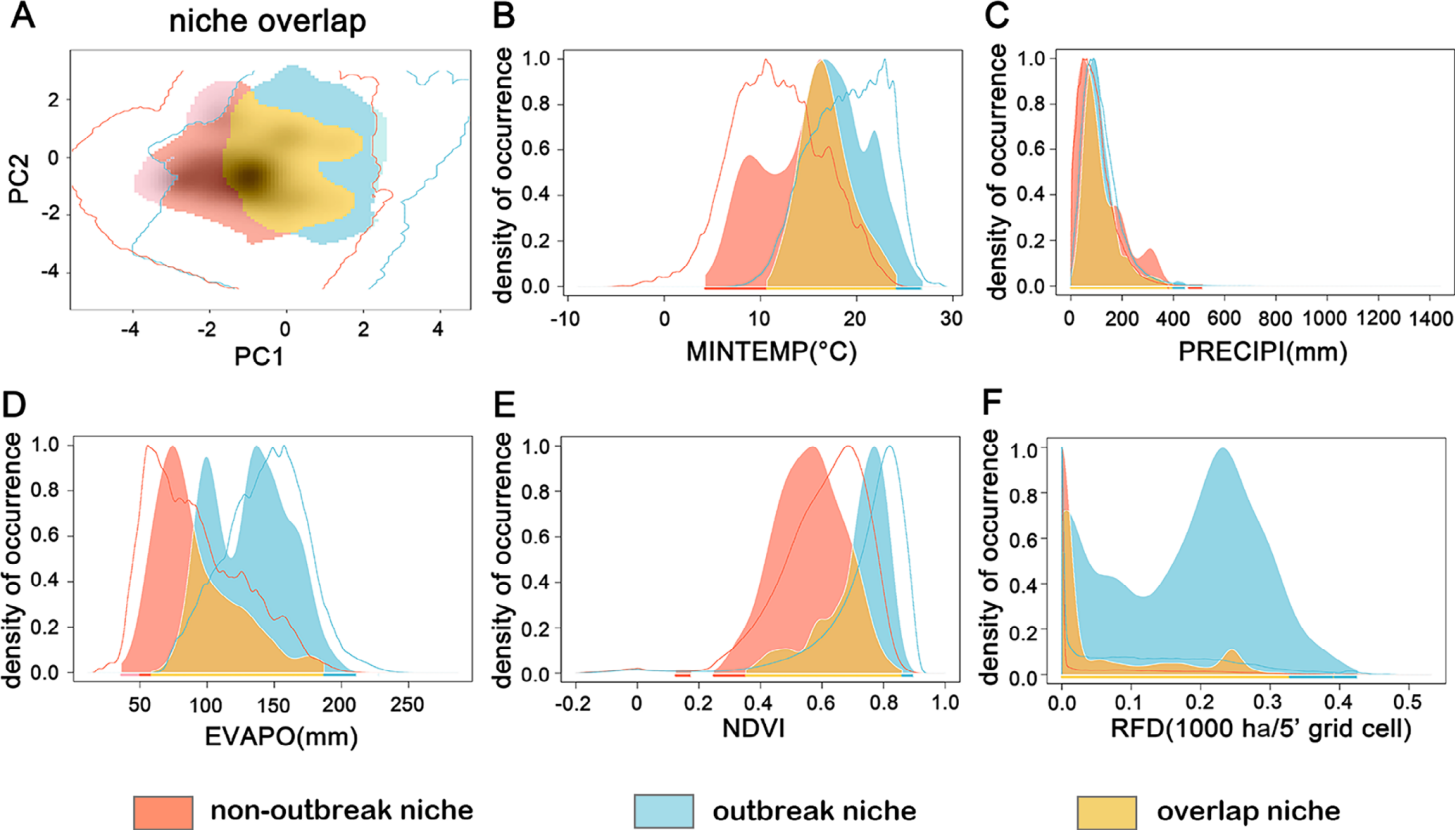
Niches comparison between fall armyworm (FAW) outbreak months (June to September) and non‐outbreak months (remaining months of the year). Red represents the unique niche during non‐outbreak months, blue represents the unique niche during outbreak months, and yellow indicates the overlapping niche between the two periods. (A) PCA analysis of niche similarity between outbreak and non‐outbreak months. The contour lines represent the available environmental space for FAW during the two periods. The colored areas indicate the niches occupied by FAW. Areas with high transparency represent non‐analogous environmental conditions. The gray‐shaded area beneath the colored areas represents the density distribution of species occurrence. (B) Niche occupancy along each environmental variable gradient. The contour lines represent the available environmental space for FAW at a single environmental level during the two periods. The colored areas indicate the niches occupied by FAW at a single environmental level. For the available environmental space, the y‐axis represents environmental density, while for the occupied niche, the y‐axis represents FAW occurrence density. Both are rescaled to a maximum value of 1. The panels illustrate the relationship between niche dynamics and different environmental variables: Minimum temperature (MINTEMP); (C) Precipitation (PRECIPI); (D) Potential evapotranspiration (EVAPO); (E) Normalized Difference Vegetation Index (NDVI); and (F) Rainfed corn cultivation area (RFD).

Comparison between the observed ecological niche in June–September (outbreak months with better model predictions and more presence points) and other months (non‐outbreak months with poorer model predictions and fewer presence points) are shown in Fig. 3. Niche dynamic indices describe the extent of overlap and mismatch between the analogous environmental conditions of outbreak and non‐outbreak months. The stable niche is 0.68 (the proportion of the overlapping niche within the non‐outbreak niche, *i.e*., yellow area / (yellow area + red area)), the expanded niche is 0.32 (the proportion of the non‐overlapping portion within the non‐outbreak niche, *i.e*., red area / (yellow area + red area)), and the unfilled niche is 0.22 (the proportion of the non‐overlapping portion within the outbreak niche, *i.e*., blue area / (yellow area + blue area)) (Fig. 3(A)). This suggests that FAW populations during ‘outbreak’ months occupy different environmental conditions compared to those during ‘non‐outbreak’ months. The relationship between niche dynamics and individual environmental variables suggests that, except for precipitation, the environmental conditions occupied by FAW differ across all variables between the two periods. (Fig. 3(B)–(F)). During outbreak months, FAW tends to occupy areas with warmer temperatures, higher evapotranspiration, greater vegetation cover, and more extensive corn cultivation (Fig. 3(B)–(F)), and this dynamic shift is closely associated with seasonal climatic changes and phenological variations.

### 3.3 Seasonal habitat prediction in the central and eastern USA

**Figure 4:**
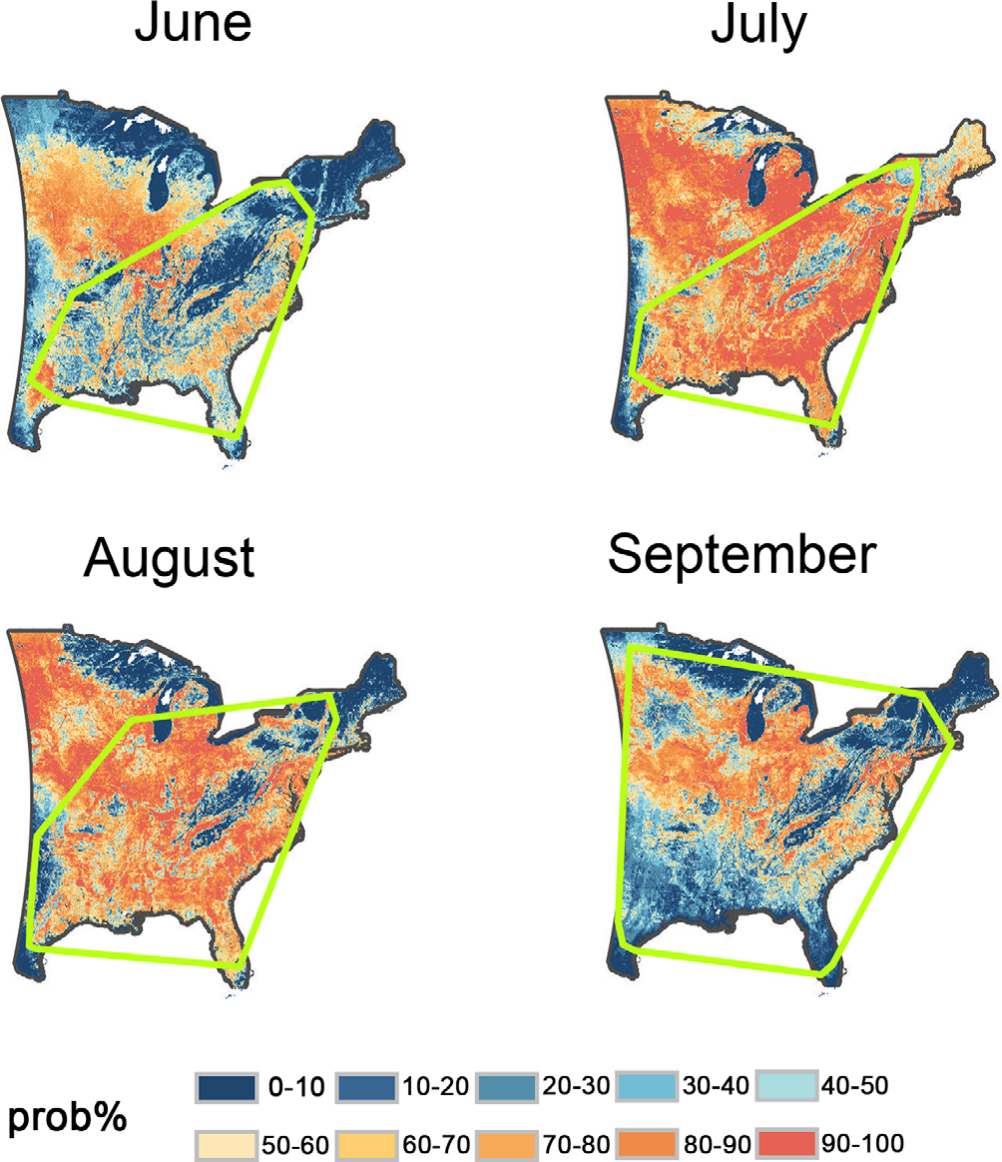
The predicted probability of fall armyworm (FAW) occurrence from June to September in the central and eastern USA. The probability of occurrence ranges from 0% (blue) to 100% (red). The green contour lines represent the minimum convex polygons enclosing the presence points of each month. Predictions were generated in the Mollweide projection (meter‐based) and reprojected to WGS84 (degree‐based) for clearer presentation, resulting in slight curvature along the western boundary due to projection differences.

The potential habitats predicted by RSEP models varied across months. During the hot summer (June to August), the potential distribution of FAW gradually expanded, with its distribution in June mainly concentrated in the southern and central regions but expanding northward in July and August to cover most areas shown on the map (Fig. 4). In September, as temperatures dropped and host plants became less available, the suitable range contracted toward the central regions (Fig. 4).

### 3.4 Relationship between FAW seasonal habitat suitability and environmental variables

The environmental variables retained through AIC stepwise selection varied by month (season) and the pseudo‐absence data used (Table 1). In the cold winter months (December, January, February), minimum temperature was the only common variable across all sub‐models (Table 1). In the gradually warming spring months (March, April, May), environmental variables related to host plants, such as NDVI and rainfed corn cultivation area, were included in all sub‐models (Table 1). With the arrival of the hot summer months (June, July, August), minimum temperature was rarely selected in the sub‐models, while precipitation and potential evapotranspiration were included in all sub‐models (Table 1). In the cool autumn months (September, October, November), minimum temperature reappeared in all sub‐models, while rainfed corn cultivation area was excluded (Table 1). Overall, the key environmental drivers of FAW potential distribution exhibit pronounced seasonal patterns: temperature plays a dominant role during the cool seasons, whereas precipitation, evapotranspiration, and host plant‐related variables are more important during the warm seasons.

**Table 1:** Environmental variable statistics of the models made with RSEP

Season/month	Common environmental variables	Frequency of each variable across all sub‐models				
		MINTEMP	PRECIPI	EVAPO	NDVI	RFD
Winter	MINTEMP	5	2	3	1	0
Spring	MINTEMP, NDVI, RFD	5	2	3	5	5
June	NDVI, RFD	3	2	3	5	5
July	EVAPO, NDVI, RFD	2	4	5	5	5
August	PRECIPI, EVAPO, NDVI, RFD	1	5	5	5	5
September	MINTEMP, NDVI, RFD	5	3	4	5	5
October	MINTEMP, PRECIPI, EVAPO, NDVI	5	5	5	5	4
November	MINTEMP, EVAPO, NDVI	5	2	5	5	0

We extracted common environmental variables and each variable's occurrence frequency across all five sub‐models made with different pseudo‐absence data for the same month (season). The abbreviations for the environmental variables are as follows: MINTEMP, minimum temperature; PRECIPI, precipitation; EVAPO, potential evapotranspiration; NDVI, normalized difference vegetation index; RFD, rainfed corn cultivation area.

**Figure 5:**
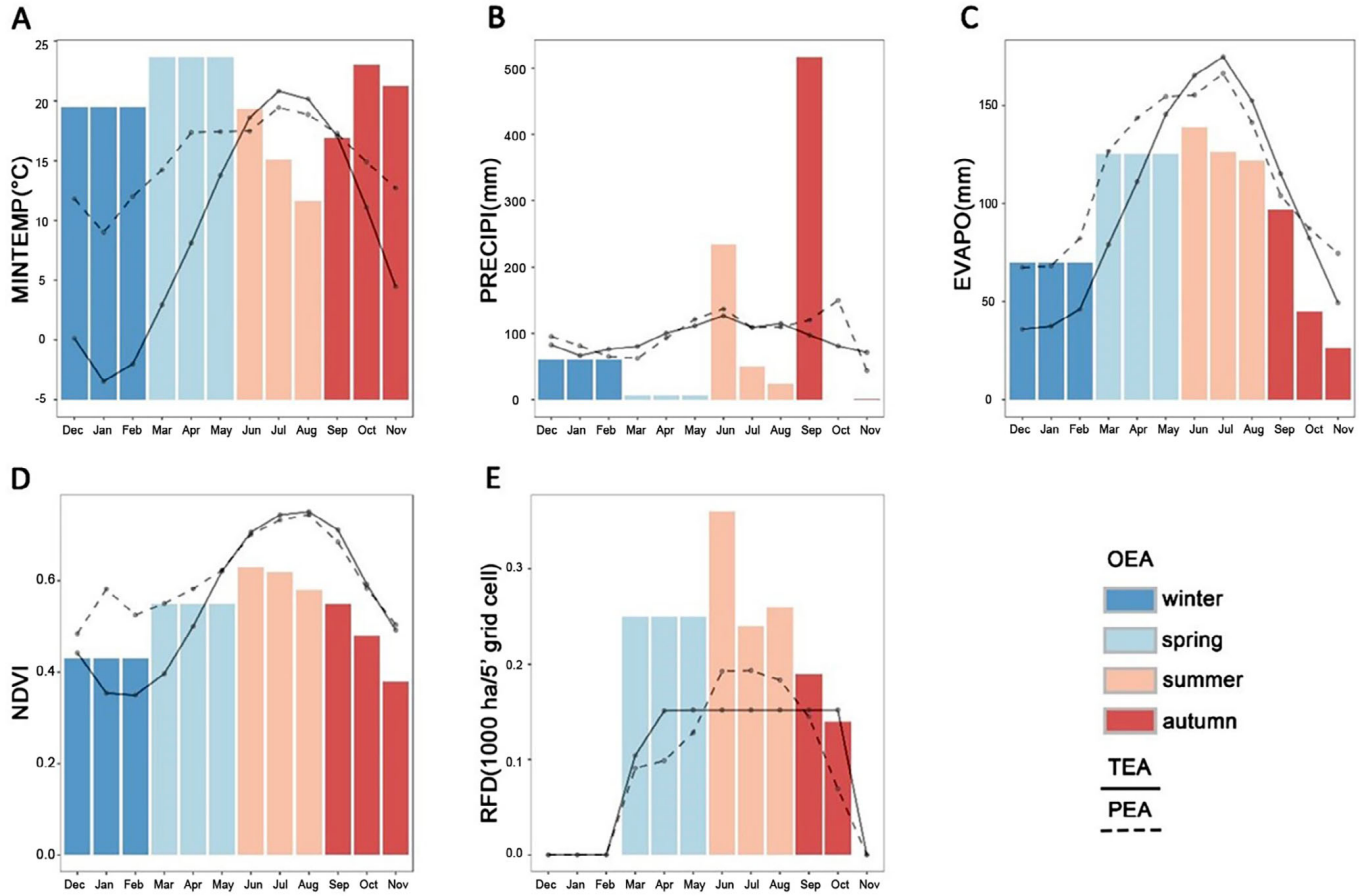
Comparison of the optimal environmental value average (OEA), the total average environmental value across the central and eastern USA (TEA), and the average environmental value at presence points (PEA). Bars represent OEA, solid lines represent TEA, and dashed lines represent PEA. Comparisons are made for (A) Minimum temperature (MINTEMP), (B) Precipitation (PRECIPI), (C) Potential evapotranspiration (EVAPO), (D) NDVI and (E) Rainfed corn cultivation area (RFD).

Across the central and eastern USA, minimum temperature followed a bell‐shaped seasonal pattern, peaking at 20°C in July. At FAW presence points, however, seasonal variation in minimum temperature was less pronounced, and the optimal values from the sub‐models decreased during the summer months, reaching 12°C in August (Fig. 5(A)). For potential evapotranspiration and NDVI, the environmental conditions in the central and eastern USA, the mean values of the presence points, and the optimal values of sub‐models all exhibited a bell‐shaped trend, peaking in summer. (Fig. 5(C), (D)).

The area of rainfed corn cultivation in the central and eastern USA peaks between May and October and decreases to zero after harvest in November. At the presence points, rainfed corn cultivation followed a similar pattern, but exceeds the average level in the central and eastern USA in summer. This suggests that FAW is preferentially found in areas of particularly high rainfed corn cultivation. The optimal area of rainfed corn cultivation increases in summer, reaching a peak in June and then declines (Fig. 5(E)). There was no clear pattern of variation in precipitation across the year, though FAW seems to experience the rainiest conditions in September (Fig. 5(B)). The fluctuations in the optimal values of each environmental variable indicate that the environmental space occupied by FAW shifts with the seasons.

## 4 DISCUSSION

Understanding the spatial and seasonal variations of agricultural pests is crucial for determining outbreak timings and implementing preventative measures. SDM methods have been widely used to predict pest distributions under current and future climate conditions, and to understand the environmental factors affecting pest distributions.^7^, ^9^, ^55^ Currently, most SDMs predict the year‐round distribution of species. However, many pests, such as FAW studied in this paper, exhibit highly seasonal migration patterns. In this study, we constructed SDMs separately for each month (whenever sufficient presence points existed for the month) or season to predict the spatiotemporal distribution of FAW in the central and eastern USA and used niche analysis to examine whether FAW's environmental conditions vary across seasons. The results showed that the model had higher predictive ability from June to September. The corresponding predictions indicated that the suitable habitat for FAW expanded rapidly in summer (June to August) and began to contract in September. In contrast, the model's predictive ability was lower for other months or seasons. Seasonally driven environmental limits led to differences in the environmental conditions occupied by FAW between outbreak months (June to September) and non‐outbreak months (the remaining months). These constraints also caused the key environmental variables influencing FAW occurrence, as well as the optimal environmental values, to vary with seasonal changes.

The presence points range of FAW, along with the SDM predictions from June to September, indicate that during summer and early autumn, FAW's suitable habitats at their widest extent nearly cover the entire central and eastern USA. This suggests that if FAW successfully reaches all suitable habitats during this period, the entire central and eastern USA could face a high risk of outbreaks. The key factor determining whether FAW can reach these habitats is the presence of favorable wind patterns that facilitate their migration. Previous studies have shown that, following three successive generations of downwind migration, FAW can travel up to 1700 km from the southernmost regions of the USA (particularly Texas and Florida) to as far north as southern Canada.^13^, ^18^, ^56^ Similarly, our collected presence points include individuals that reached the northernmost regions of the USA during outbreak months. This indicates that strong wind patterns capable of supporting the long‐distance migration of FAW across the entire USA do exist in the atmosphere. Therefore, as long as wind direction and speed are favorable, FAW may invade any part of the central and eastern USA, triggering widespread outbreaks and posing a significant threat to agriculture.

Although our models predict high FAW suitability in most of the midwestern USA during outbreak months, the actual presence records from this region remain limited, likely due to incomplete data collection; however, we believe the model predictions are accurate. A previous study on FAW population growth model in the USA also indicated that a large number of FAW could accumulate in this region during outbreak months.^13^, ^56^

From June to September, the predictive ability of the models is higher than in other months, possibly because the presence points collected during this outbreak period are both greater in number and more widely distributed. These extensive presence points provide a solid foundation for modeling, enabling the capture of a relatively complete ecological niche.^57^ In contrast, during non‐outbreak periods, the number of collected points is relatively small, and many of them are restricted to the year‐round habitat in the southern USA (Fig. 1). This data limitation makes it challenging for SDMs to accurately characterize the complex relationship between the distribution patterns of FAW and environmental variables across large‐scale regions such as the central and eastern USA, ultimately reducing the models' predictive accuracy.^57^, ^58^ To improve the predictive ability of SDMs during non‐outbreak months, the frequency of field surveys in FAW seasonal habitats should be increased during this period to enhance FAW detection rates and ensure that the models obtain sufficient presence points.

It is worth noting that in outbreak months, the model's specificity is lower than its sensitivity, meaning that while the model successfully identifies most FAW occurrences, it also tends to incorrectly predict some areas that are not actually suitable for FAW survival as suitable habitats, leading to an overestimation of FAW's habitat range.^59^ Species distribution models generally assume that the study area has been systematically or randomly sampled. However, during outbreak months, many easily accessible areas were extensively surveyed, resulting in a clustered distribution of species occurrence data. This may lead to commission errors (false positives), where the model incorrectly predicts the presence of a species.^60^, ^61^

The common environmental variables in all sub‐models of the same month (season) change with the month (season), indicating that the environmental factors that play a decisive role in FAW distribution change over time throughout the year. In summer, much of the central and eastern USA is warm and suitable for FAW reproduction (Fig. S3), so the minimum temperature (MINTEMP) no longer constrains FAW distribution during June to August and is excluded from the models' common variables. In the subsequent fall and winter, when temperatures drop (Fig. S3), the cold regions unsuitable for FAW survival expand, and minimum temperature is reintroduced into the common variables. Precipitation (PRECIPI) and potential evapotranspiration (EVAPO) are included in the common variables during the months when the model predicts the most severe FAW outbreak (July and August). Humidity is a key environmental factor influencing insect survival, growth, and reproduction.^62^ Since precipitation and evapotranspiration jointly determine environmental humidity, they might serve as proxy variables for humidity in the SDM. During summer, high temperatures accelerate surface water loss, while drought caused by low humidity disrupts the water balance in insects and negatively impacts their survival.^62^ Additionally, drought can reduce host plant quality, indirectly affecting FAW population viability.^63^ The decrease in the optimal minimum temperature values in the model for July and August also supports our viewpoint (Fig. 5(A)). In the hot months (July and August), humidity becomes a key environmental factor influencing FAW survival. Therefore, incorporating precipitation and evapotranspiration into the SDM helps improve the model's accuracy. In contrast, during other months, lower temperatures reduce the likelihood of high temperature‐induced droughts. As a result, precipitation and evapotranspiration do not play a major role in determining survival rate during these months. NDVI and rainfed corn cultivation area (RFD), which are related to vegetation cover, are included in the majority of seasonal models, underscoring the irreplaceable role of food resources in FAW survival.

It is worth noting that although FAW has a wide range of host plants, the vast majority of current monitoring efforts have been concentrated in agricultural areas, particularly in cornfields. As a result, FAW populations associated with alternative host plants may not have been fully documented, potentially introducing a sampling bias. In light of this practical limitation, our modeling in this study incorporated only corn cultivation data and did not account for the full range of potential host plants. This data limitation may have contributed to some degree of inaccuracy in the model predictions. Moreover, the corn cultivation data used in this study did not include regions where sweet corn is grown during winter and early spring in the USA However, FAW populations in southern Florida rely on this sweet corn production to persist through winter, showing opposite seasonal patterns from the seasonal habitat—high in winter, low in summer.^64^, ^65^ This unique overwintering pattern is important for understanding FAW migration and identifying potential year‐round habitats in invaded areas. It provides useful reference for predicting overwintering hotspots and migration routes in Europe, such as North Africa, southern Spain, southern Greece, or Cyprus.

Through niche analysis, we found substantial differences in the environmental spaces occupied by FAW during outbreak and non‐outbreak months. Excluding precipitation, both the environmental range occupied by FAW during outbreak months and the available environmental range are greater than those during non‐outbreak months (Fig. 3). Correspondingly, the optimal environmental conditions for FAW occurrence in the model vary by month, with the trends in optimal EVAPO, NDVI, and RFD values consistent with overall environmental changes in the central and eastern USA (Fig. 5). These two results together indicate that the environmental space occupied by FAW in different seasons is influenced by variations in available environmental conditions across different months. The northern part of the central and eastern USA is cold and dry in winter and warm and humid in summer, with vegetation cover and cropping patterns adapting to climatic changes (Fig. S3). The environmental values at FAW presence points, pseudo‐absence points, and background areas required for niche analysis reflect the environmental characteristics of their respective months and are constrained by the threshold values of the environmental variables for those months. Such constrained data will result in niche analyses indicating that the ecological niche seems to shift across different time periods and cause the SDMs for different months to generate unique relationships between FAW distribution and environment, as reflected in the fluctuation of common environmental variable types and optimal environmental values across models for different months (seasons).

Most studies currently construct a single seasonal model using all presence points throughout the year.^66^, ^67^, ^68^ This approach assumes that the species does not undergo niche shifts between seasons and that the relationship between environmental factors and species distribution remains consistent across all seasons. However, our findings do not support this assumption. To ensure that SDMs accurately capture these seasonal niche variations, it is essential to construct separate SDMs for each season, and match distributions to the appropriate months or seasons.

Although our findings suggest that FAW occupies different ecological niches in different seasons, the primary reason for this niche differentiation might be due to phenological effects. The varying environmental conditions available at different times of the year could lead to the seasonal niches we captured, which only represent portions of the species' full ecological niche rather than a true change in the species' preference for environmental conditions.^6^, ^69^ In this study, we do not have sufficient evidence to confirm that FAW exhibits phenotypic plasticity in response to seasonal environmental changes, such as differences in tolerance to extreme temperatures or host plant preferences. To exclude the influence of seasonal environmental thresholds and investigate whether FAW's environmental preferences change across different seasons, we suggest conducting controlled laboratory experiments with samples collected in different seasons. This would allow us to examine whether FAW's survival response to a single variable remains consistent across different seasons under the same levels of other environmental factors. Existing research has already shown that FAW larvae collected in winter exhibit higher cold tolerance compared to those collected in summer, indicating that FAW possesses phenotypic plasticity in response to temperature conditions.^70^ This provides evidence supporting the true niche shift in FAW as an active adaptation to seasonal environmental conditions.

In the model construction process, we used the RS and RSEP methods as candidate approaches for selecting pseudo‐absence points, with the latter ultimately chosen due to its improved predictive ability. Specifically, the RS method randomly generated many pseudo‐absence points around presence points, causing pseudo‐absence points to potentially appear in areas suitable for species distribution. In contrast, the RSEP method sampled in areas different from the presence point environment, causing the pseudo‐absence points to be more concentrated in non‐suitable survival areas. As a result, the fitted model becomes stronger and offers higher resolution, characterized by a reduced frequency of ambiguous predictions (close to 0.5) and an increased frequency of confident predictions (approaching 0 or 1).^71^, ^72^

This study highlights that key environmental factors influencing pest survival are affected by seasonal environmental changes. Therefore, constructing independent SDMs for each season can improve prediction accuracy. The seasonal niche variations observed in this study primarily result from capturing different portions of the full niche in different seasons. However, whether these variations indicate a shift of the full niche in FAW as an adaptation to seasonal environmental conditions requires further validation. To address this, we recommend controlled laboratory experiments to systematically assess FAW's tolerance to various environmental factors across different seasons. This research enhances our understanding of pest environmental adaptability and provides a scientific basis for more accurate predictions of pest outbreaks, ultimately contributing to crop pest management in North America and globally.

## CONFLICT OF INTEREST

The authors declare no conflicts of interest associated with any aspect of this study.

## Supporting information


**Figure S1.** The Pearson correlation matrices assess collinearity among environmental variables for modeling the habitat suitability of FAW, presented across eight images, each corresponding to a specific month (season). The correlation coefficients were rounded to two decimal places. Darker shades indicate high collinearity between variables, while lighter shades represent low collinearity. The abbreviations for the environmental variables are as follows: MAXTEMP ‐ maximum temperature; MINTEMP ‐ minimum temperature; PRECIPI ‐ precipitation; EVAPO ‐ potential evapotranspiration; VAPOR ‐ vapor pressure deficit; NDVI ‐ normalized difference vegetation index; RFD ‐ rainfed corn cultivation areas.
**Figure S2.** Comparison of Sensitivity (SEN) and Specificity (SPE) of seasonal sub‐models in RS and RSEP models with the threshold at (A) SEN = SPE and (B) SEN = 0.95.
**Figure S3.** Environmental average rasters for all months. Different colors represent the threshold intervals of different environmental variables, with specific threshold interval information provided in Table S1. The abbreviations for the environmental variables are as follows: MINTEMP ‐ minimum temperature; PRECIPI ‐ precipitation; EVAPO ‐ potential evapotranspiration; NDVI ‐ normalized difference vegetation index; RFD ‐ rainfed corn cultivation areas.
**Table S1.** The correspondence between the threshold intervals of environmental variables and the color index. The abbreviations for the environmental variables are as follows: MINTEMP ‐ minimum temperature; PRECIPI ‐ precipitation; EVAPO ‐ potential evapotranspiration; NDVI ‐ normalized difference vegetation index; RFD ‐ rainfed corn cultivation areas.

**Table S2.** Some data providers require us to keep the exact locations of presence points confidential. Therefore, instead of disclosing the original coordinates of presence points, we convert them to the center coordinates of the corresponding environmental grid.

## Data Availability

The data that supports the findings of this study are available in the supplementary material of this article.
