# Supplementary material for: Predicting the seasonal distribution of fall armyworm in North America using species distribution models
Source: Pest Manag Sci. 2025 Sep 18;82(1):560–70. doi: 10.1002/ps.70217 (PMC12713716; doi:10.1002/ps.70217)
Supplement: Supplementary file 1 — Figure S1. The Pearson correlation matrices assess collinearity among environmental variables for modeling the habitat suitability of FAW, presented across eight images, each corresponding to a specific month (season). The correlation coefficients were rounded to two decimal places. Darker shades indicate high collinearity between variables, while lighter shades represent low collinearity. The abbreviations for the environmental variables are as follows: MAXTEMP ‐ maximum temperature; MINTEMP ‐ minimum temperature; PRECIPI ‐ precipitation; EVAPO ‐ potential evapotranspiration; VAPOR ‐ vapor pressure deficit; NDVI ‐ normalized difference vegetation index; RFD ‐ rainfed corn cultivation areas. Figure S2. Comparison of Sensitivity (SEN) and Specificity (SPE) of seasonal sub‐models in RS and RSEP models with the threshold at (A) SEN = SPE and (B) SEN = 0.95. Figure S3. Environmental average rasters for all months. Different colors represent the threshold intervals of different environmental variables, with specific threshold interval information provided in Table S1. The abbreviations for the environmental variables are as follows: MINTEMP ‐ minimum temperature; PRECIPI ‐ precipitation; EVAPO ‐ potential evapotranspiration; NDVI ‐ normalized difference vegetation index; RFD ‐ rainfed corn cultivation areas. Table S1. The correspondence between the threshold intervals of environmental variables and the color index. The abbreviations for the environmental variables are as follows: MINTEMP ‐ minimum temperature; PRECIPI ‐ precipitation; EVAPO ‐ potential evapotranspiration; NDVI ‐ normalized difference vegetation index; RFD ‐ rainfed corn cultivation areas. [file PS-82-560-s002.docx]

**Supplemental Information**

**Predicting the seasonal distribution of fall armyworm in North America using species distribution models**

Fan-Qi Gao^1^, Robert L. Meagher^2^, Rodney N. Nagoshi^2^, Jason W. Chapman ^1,3^, Regan Early ^1^

^1^ Centre for Ecology and Conservation, University of Exeter, Penryn, Cornwall TR10 9FE, United Kingdom

^2^ Center for Medical, Agricultural and Veterinary Entomology, Agricultural Research Service, U.S. Department of Agriculture, Gainesville, FL 32608, United States

^3^ Department of Entomology, Nanjing Agricultural University, Nanjing 210095, China

* To whom correspondence may be addressed. Email: [fg362@exeter.ac.uk](mailto:fg362@exeter.ac.uk)

**
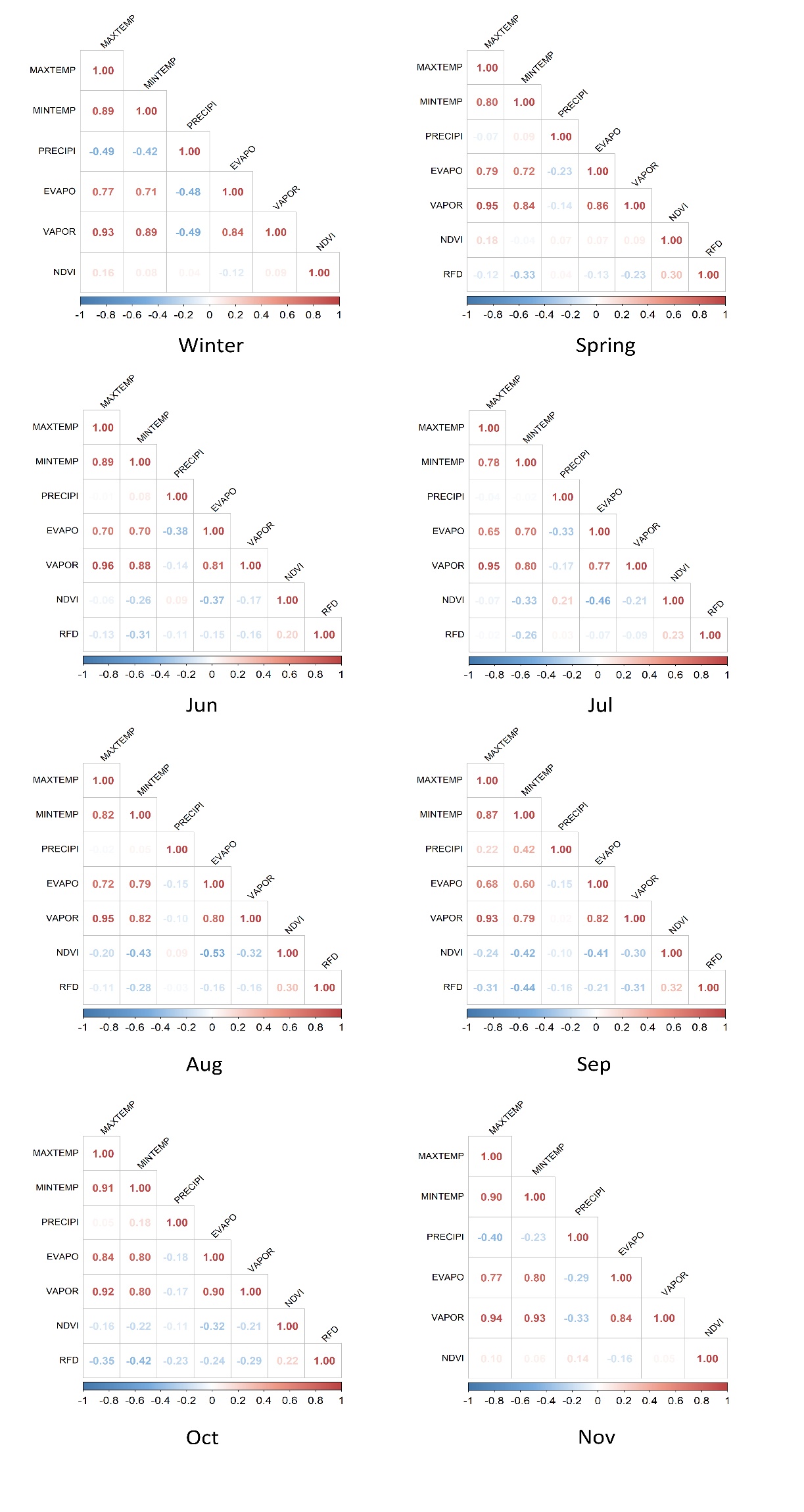
**

**Figure S1** The Pearson correlation matrices assess collinearity among environmental variables for modeling the habitat suitability of FAW, presented across 8 images, each corresponding to a specific month (season). The correlation coefficients were rounded to two decimal places. Darker shades indicate high collinearity between variables, while lighter shades represent low collinearity. The abbreviations for the environmental variables are as follows: MAXTEMP - maximum temperature; MINTEMP - minimum temperature; PRECIPI - precipitation; EVAPO - potential evapotranspiration; VAPOR - vapor pressure deficit; NDVI - normalized difference vegetation index; RFD - rainfed corn cultivation areas.


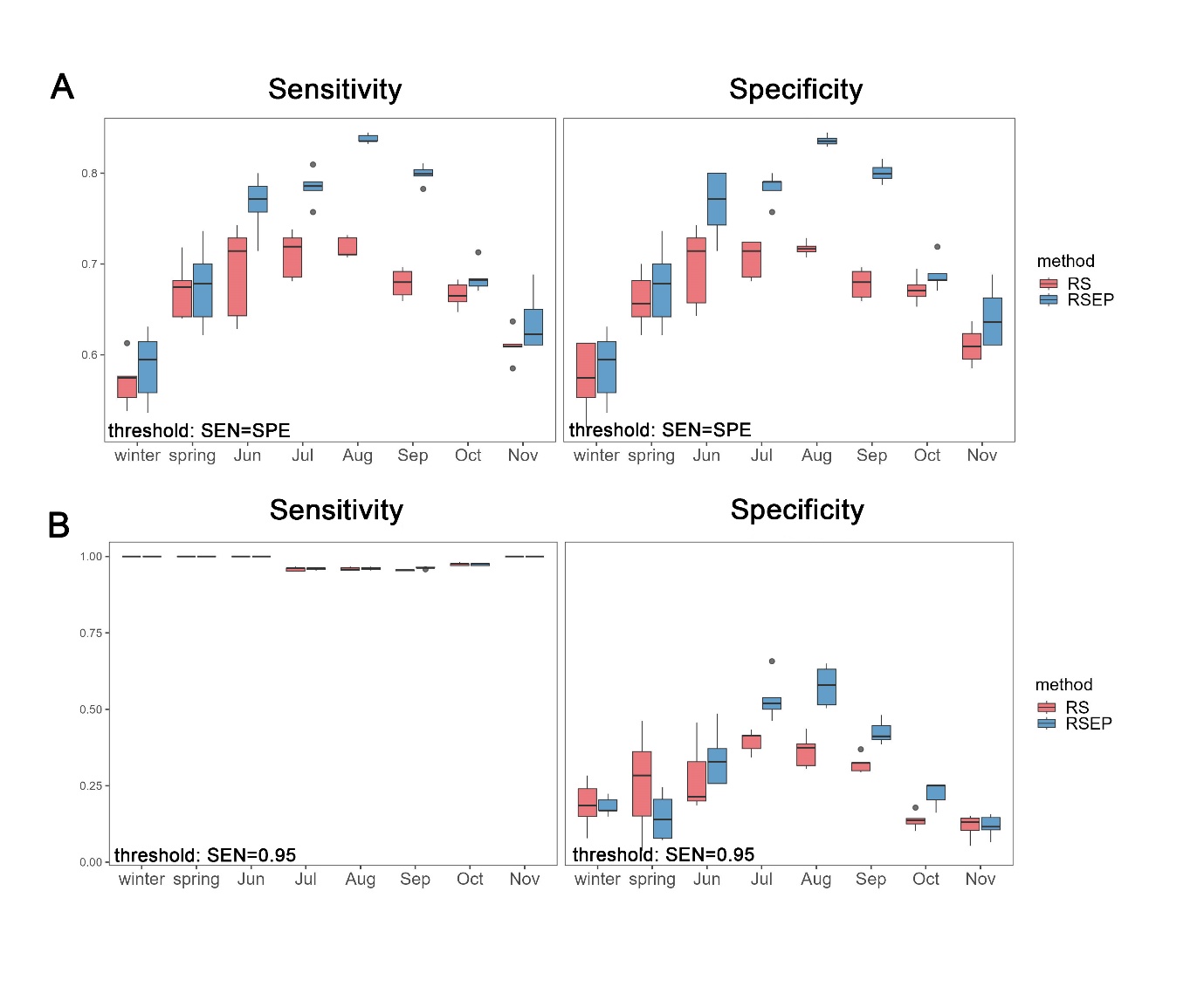


**Figure S2** Comparison of Sensitivity (SEN) and Specificity (SPE) of seasonal sub-models in RS and RSEP models with the threshold at (A) SEN = SPE and (B) SEN = 0.95


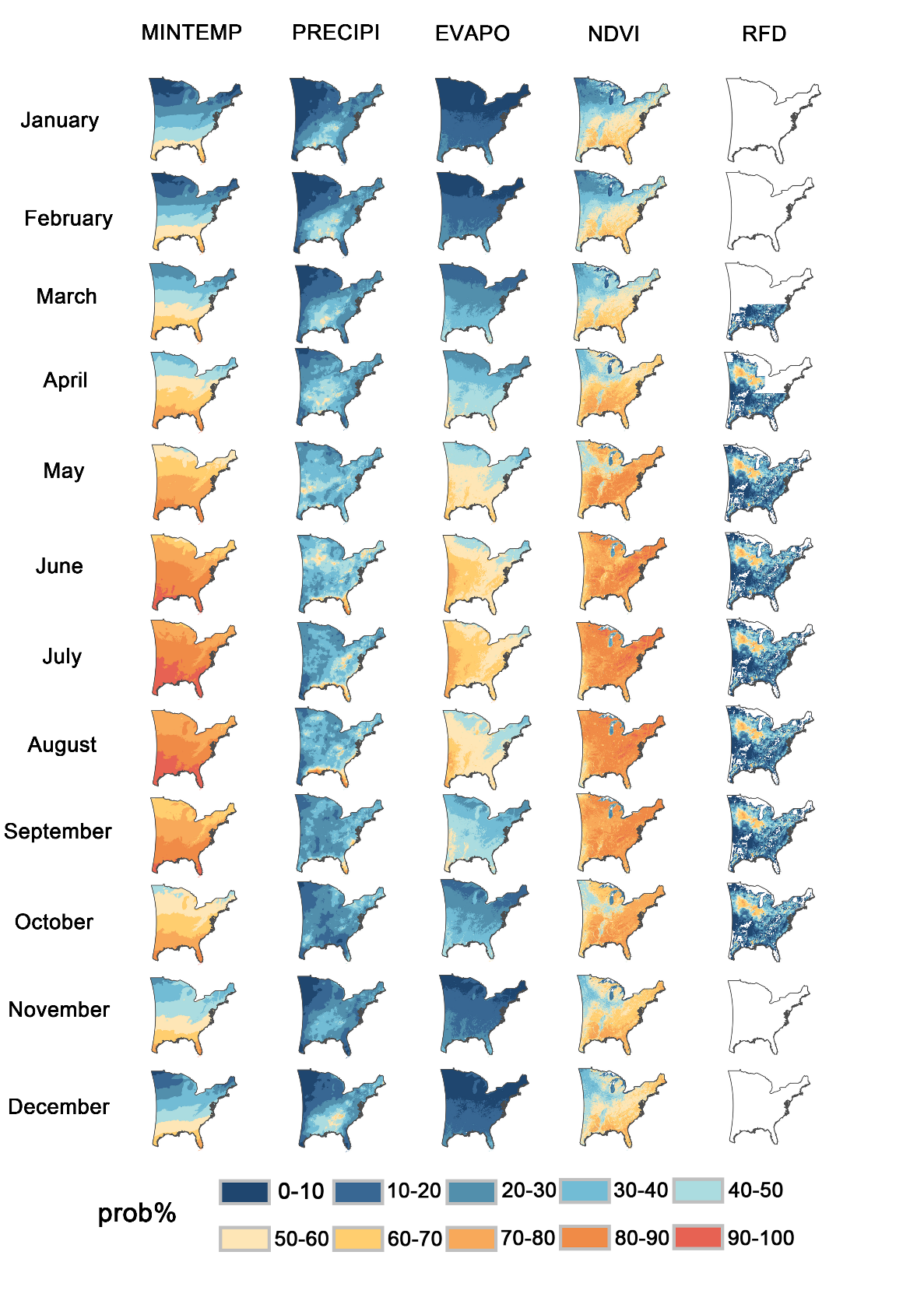


**Figure S3** Environmental average rasters for all months. Different colors represent the threshold intervals of different environmental variables, with specific threshold interval information provided in Table S1. The abbreviations for the environmental variables are as follows: MINTEMP - minimum temperature; PRECIPI - precipitation; EVAPO - potential evapotranspiration; NDVI - normalized difference vegetation index; RFD - rainfed corn cultivation areas.

**Table S1** The correspondence between the threshold intervals of environmental variables and the color index. The abbreviations for the environmental variables are as follows: MINTEMP - minimum temperature; PRECIPI - precipitation; EVAPO - potential evapotranspiration; NDVI - normalized difference vegetation index; RFD - rainfed corn cultivation areas.

| **color index** | **MINTEMP**  **(**°C**)** | **PRECIPI**  **(mm)** | | **EVAPO**  **(mm)** | **NDVI** | **RFD**  **1,000 ha per 5' grid** |
| --- | --- | --- | --- | --- | --- | --- |
| 1 | -18 to -13.4 | | 7 to 38 | 6 to 33.1 | -0.2 to -0.08 | 0 to 0.06 |
| 2 | -13.4 to -8.8 | | 38 to 69 | 33.1 to 60.2 | -0.08 to 0.04 | 0.06 to 0.12 |
| 3 | -8.8 to -4.2 | | 69 to 100 | 60.2 to 87.3 | 0.04 to 0.16 | 0.12 to 0.18 |
| 4 | -4.3 to 0.4 | | 100 to 131 | 87.3 to 114.4 | 0.16 to 0.28 | 0.18 to 0.24 |
| 5 | 0.4 to 5 | | 131 to 162 | 114.4 to 141.5 | 0.28 to 0.4 | 0.24 to 0.3 |
| 6 | 5 to 9.6 | | 162 to 193 | 141.5 to 168.6 | 0.4 to 0.52 | 0.3 to 0.36 |
| 7 | 9.6 to 14.2 | | 193 to 224 | 168.6 to 195.7 | 0.52 to 0.64 | 0.36 to 0.42 |
| 8 | 14.2 to 18.8 | | 224 to 255 | 195.7 to 222.8 | 0.64 to 0.76 | 0.42 to 0.48 |
| 9 | 18.8 to 23.4 | | 255 to 286 | 222.8 to 249.9 | 0.76 to 0.88 | 0.48 to 0.54 |
| 10 | 23.4 to 28 | | 286 to 317 | 249.9 to 277 | 0.88 to 1 | 0.54 to 0.6 |
